# Transmission of SARS-COV-2 Infections in Households — Tennessee and Wisconsin, April–September 2020

**DOI:** 10.15585/mmwr.mm6944e1

**Published:** 2020-11-06

**Authors:** Carlos G. Grijalva, Melissa A. Rolfes, Yuwei Zhu, Huong Q. McLean, Kayla E. Hanson, Edward A. Belongia, Natasha B. Halasa, Ahra Kim, Carrie Reed, Alicia M. Fry, H. Keipp Talbot

**Affiliations:** ^1^Vanderbilt University Medical Center, Nashville, Tennessee; ^2^CDC COVID-19 Response Team; ^3^Marshfield Clinic Research Institute, Marshfield, Wisconsin.

*On October 30, 2020, this report was posted online as an *MMWR* Early Release.*

Improved understanding of transmission of SARS-CoV-2, the virus that causes coronavirus disease 2019 (COVID-19), within households could aid control measures. However, few studies have systematically characterized the transmission of SARS-CoV-2 in U.S. households (*1*). Previously reported transmission rates vary widely, and data on transmission rates from children are limited. To assess household transmission, a case-ascertained study was conducted in Nashville, Tennessee, and Marshfield, Wisconsin, commencing in April 2020. In this study, index patients were defined as the first household members with COVID-19–compatible symptoms who received a positive SARS-CoV-2 reverse transcription–polymerase chain reaction (RT-PCR) test result, and who lived with at least one other household member. After enrollment, index patients and household members were trained remotely by study staff members to complete symptom diaries and obtain self-collected specimens, nasal swabs only or nasal swabs and saliva samples, daily for 14 days. For this analysis, specimens from the first 7 days were tested for SARS-CoV-2 using CDC RT-PCR protocols.^†^ A total of 191 enrolled household contacts of 101 index patients reported having no symptoms on the day of the associated index patient’s illness onset, and among these 191 contacts, 102 had SARS-CoV-2 detected in either nasal or saliva specimens during follow-up, for a secondary infection rate of 53% (95% confidence interval [CI] = 46%–60%). Among fourteen households in which the index patient was aged <18 years, the secondary infection rate from index patients aged <12 years was 53% (95% CI = 31%–74%) and from index patients aged 12–17 years was 38% (95% CI = 23%–56%). Approximately 75% of secondary infections were identified within 5 days of the index patient’s illness onset, and substantial transmission occurred whether the index patient was an adult or a child. Because household transmission of SARS-CoV-2 is common and can occur rapidly after the index patient’s illness onset, persons should self-isolate immediately at the onset of COVID-like symptoms, at the time of testing as a result of a high risk exposure, or at the time of a positive test result, whichever comes first. Concurrent to isolation, all members of the household should wear a mask when in shared spaces in the household.^§^

The data presented in this report are from an ongoing, CDC-supported study of household transmission of SARS-CoV-2 in Nashville, Tennessee and Marshfield, Wisconsin, and include households enrolled during April–September 2020.^¶^ Households were eligible if the index patient had symptom onset <7 days before household enrollment and the household included at least one other person who was not symptomatic at the time of the index patient’s illness onset and was thus deemed to be at risk. Characteristics of the index patients, household members, and their interactions were ascertained using Research Electronic Data Capture (REDCap),** an online application for data collection, or through paper-based surveys. The 7-day secondary infection rate was calculated by dividing the number of laboratory-confirmed SARS-CoV-2 infections identified during the 7-day follow-up period by the number of household members at risk per 100 population.^††^ Because saliva samples are considered an emerging diagnostic approach but are not yet standard for SARS-CoV-2 detection (*2*), secondary infection rates were also calculated using positive test results from nasal swab specimens only. To account for household members possibly having been infected when the index case became ill, secondary infections rates were also conservatively calculated excluding household members who had positive test results at enrollment. The study protocol was reviewed and approved by the Vanderbilt University Medical Center’s and Marshfield Clinic Research Institute’s Institutional Review Board, and was conducted consistent with applicable federal law and CDC policy.^§§^

For this analysis, 101 households (including 101 index patients and 191 household members) were enrolled and completed ≥7 days of follow-up. The median index patient age was 32 years (range = 4–76 years; interquartile range [IQR] = 24–48 years); 14 (14%) index patients were aged <18 years, including five aged <12 years and nine aged 12–17 years. Among index patients, 75 (74%) were non-Hispanic White, eight (8%) were non-Hispanic persons of other races, and 18 (18%) were Hispanic or Latino (Table 1). Index patients received testing for SARS-CoV-2 a median of 1 day (IQR = 1–2) after illness onset and were enrolled in the study a median of 4 days (IQR = 2–4) after illness onset.

**TABLE 1:** Characteristics of index patients with laboratory-confirmed SARS-CoV-2 infection and household members enrolled in a prospective study of SARS-CoV-2 household transmission — Tennessee and Wisconsin, April–September 2020

Characteristic	No. (%)*	
	Index patients (n = 101)	Household members (n = 191)
**Median age, yrs (IQR)**	32 (24–48)	28 (14–46)
**Age group, yrs**		
<12	5 (5)	32 (17)
12–17	9 (9)	30 (16)
18–49	65 (64)	92 (48)
≥50	22 (22)	37 (19)
**Male**	41 (41)	88 (46)
**Race/Ethnicity**		
White, non-Hispanic	75 (74)	127 (67)
Other race, non-Hispanic	8 (8)	24 (13)
Hispanic or Latino	18 (18)	40 (21)
**Underlying medical condition**		
Any	22 (22)	37 (19)
Asthma	12 (12)	24 (13)
Other chronic lung disease	0 (0)	2 (1)
Cardiovascular disease	4 (4)	7 (4)
Diabetes	4 (4)	7 (4)
Chronic renal disease	0 (0)	2 (1)
Immunocompromising condition	2 (2)	3 (2)
Smoking/Vaping^†^	2 (2)	4 (2)

**Abbreviation:** IQR = interquartile range.

* Percentages might not sum to 100% because of rounding.

^†^ Data available for 98 index cases and 166 household members.

The median number of household members per bedroom was one (IQR = 0.8–1.3). Seventy (69%) index patients reported spending >4 hours in the same room with one or more household members the day before and 40 (40%) the day after illness onset. Similarly, 40 (40%) of index patients reported sleeping in the same room with one or more household members before illness onset and 30 (30%) after illness onset.

Among all household members, 102 had nasal swabs or saliva specimens in which SARS-CoV-2 was detected by RT-PCR during the first 7 days of follow-up, for a secondary infection rate of 53% (95% CI = 46%–60%) (Table 2). Secondary infection rates based only on nasal swab specimens yielded similar results (47%, 95% CI = 40%–54%). Excluding 54 household members who had SARS-CoV-2 detected in specimens taken at enrollment, the secondary infection rate was 35% (95% CI = 28%–43%).

**TABLE 2:** Rates of secondary laboratory-confirmed SARS-CoV-2 infections among household members enrolled in a prospective study of SARS-CoV-2 household transmission — Tennessee and Wisconsin, April–September 2020

Characteristic	Laboratory-confirmed SARS-CoV-2 infections/Household members at risk	Secondary infection rate % (95% CI)*
**All household members**	102/191	53 (46–60)
**Nasal swab–positive tests only**	89/191	47 (40–54)
**RT-PCR–negative at enrollment**	48/137	35 (28–43)
**Index patient age group, yrs**		
<12	9/17	53 (31–74)
12–17	11/29	38 (23–56)
18–49	64/116	55 (46–64)
≥50	18/29	62 (44–77)
**Index patient sex**		
Female	66/108	61 (52–70)
Male	36/83	43 (33–54)
**Index patient race/ethnicity**		
White, non-Hispanic	71/139	51 (43–59)
Other race, non-Hispanic	9/17	53 (31–74)
Hispanic or Latino	22/35	63 (46–77)
**Household member age group, yrs**		
<12	18/32	57 (39–72)
12–17	14/30	47 (30–64)
18–49	54/92	59 (48–68)
≥50	16/37	43 (29–59)
**Household member sex**		
Female	52/103	50 (41–60)
Male	50/88	57 (46–67)
**Household member race/ethnicity**		
White, non-Hispanic	67/127	53 (44–61)
Other race, non-Hispanic	9/24	38 (21–57)
Hispanic or Latino	26/40	65 (50–78)
**Household size, no. of persons**		
2	26/38	68 (53–81)
3	25/41	61 (46–74)
4	18/40	45 (31–60)
≥5	33/72	46 (35–57)

**Abbreviations:** CI = confidence interval; RT-PCR = reverse transcription–polymerase chain reaction.

* Secondary infection rate, and 95% CI, estimated over 7 days of follow-up. Enrolled household members who did not report symptoms at time of illness onset in the index case-patient were considered at risk.

Forty percent (41 of 102) of infected household members reported symptoms at the time SARS-CoV-2 was first detected by RT-PCR. During 7 days of follow-up, 67% (68 of 102) of infected household members reported symptoms, which began a median of 4 days (IQR = 3–5) after the index patient’s illness onset. The rates of symptomatic and asymptomatic laboratory-confirmed SARS-CoV-2 infection among household members was 36% (95% CI = 29%–43%) and 18% (95% CI = 13%–24%), respectively.

## Discussion

In this ongoing prospective study that includes systematic and daily follow-up, transmission of SARS-CoV-2 among household members was common, and secondary infection rates were higher than have been previously reported (*1*,*3*–*7*). Secondary infections occurred rapidly, with approximately 75% of infections identified within 5 days of the index patient’s illness onset. Secondary infection rates were high across all racial/ethnic groups. Substantial transmission occurred whether the index patient was an adult or a child.

Several studies have reported estimates of household transmission, largely from contact tracing activities, with limited follow-up and testing of household members or delayed enrollment relative to index patient identification (*3*–*5*,*7*). These different approaches to ascertain infections could explain the higher secondary infection rates observed in this study relative to other estimates. In addition, other studies, particularly those conducted abroad, might have found lower secondary infection rates because of rapid isolation of patients in facilities outside households or different adoption of control measures, such as mask use, in the home (*3*–*5*,*7*,*8*).

Because prompt isolation of persons with COVID-19 can reduce household transmission, persons who suspect that they might have COVID-19 should isolate, stay at home, and use a separate bedroom and bathroom if feasible. Isolation should begin before seeking testing and before test results become available because delaying isolation until confirmation of infection could miss an opportunity to reduce transmission to others. Concurrently, all household members, including the index patient, should start wearing a mask in the home, particularly in shared spaces where appropriate distancing is not possible. Close household contacts of the index patient should also self-quarantine, to the extent possible, particularly staying away from those at higher risk of getting severe COVID-19. To complement these measures within the household, a potential approach to reduce SARS-CoV-2 transmission at the community level would involve detecting infections before onset of clinical manifestations; this would require frequent and systematic testing in the community with rapidly available results to enable prompt adoption of preventive measures. The feasibility and practicality of this approach is undergoing extensive discussion (*9*) and study. This ongoing household transmission study will provide critical data regarding the recommended timing and frequency of testing.

An important finding of this study is that fewer than one half of household members with confirmed SARS-CoV-2 infections reported symptoms at the time infection was first detected, and many reported no symptoms throughout 7 days of follow-up, underscoring the potential for transmission from asymptomatic secondary contacts and the importance of quarantine. Persons aware of recent close contact with an infected person, such as a household member, should quarantine in their homes and get tested for SARS-CoV-2.^¶¶^

The findings in this study are subject to at least four limitations. First, the initial household member who experienced symptoms was considered the index patient, but it is possible that other household members were infected concurrently but developed symptoms at different times or remained asymptomatic. Although households were enrolled rapidly, several infections among household members were already detectable at enrollment, underscoring the rapid spread of infections within households and the challenge inherent in conclusively reconstructing the transmission sequence. Second, although living in the same household might impart a high risk of acquiring infection, some infections might have originated outside the household, leading to higher apparent secondary infection rates. Third, respiratory samples were self-collected; although this might have reduced the sensitivity of detections, studies have reported performance comparable to clinician-collected samples (*10*). Finally, the families in the study might not be representative of the general U.S. population.

These findings suggest that transmission of SARS-CoV-2 within households is high, occurs quickly, and can originate from both children and adults. Prompt adoption of disease control measures, including self-isolating at home, appropriate self-quarantine of household contacts, and all household members wearing a mask in shared spaces, can reduce the probability of household transmission.

**Summary**

**What is already known about this topic?**

Transmission of SARS-CoV-2 occurs within households; however, transmission estimates vary widely and the data on transmission from children are limited.

**What is added by this report?**

Findings from a prospective household study with intensive daily observation for ≥7 consecutive days indicate that transmission of SARS-CoV-2 among household members was frequent from either children or adults.

**What are the implications for public health practice?**

Household transmission of SARS-CoV-2 is common and occurs early after illness onset. Persons should self-isolate immediately at the onset of COVID-like symptoms, at the time of testing as a result of a high risk exposure, or at time of a positive test result, whichever comes first. All household members, including the index case, should wear masks within shared spaces in the household.

## Footnotes

† https://www.fda.gov/media/134922/download.

§ https://www.cdc.gov/coronavirus/2019-ncov/if-you-are-sick/steps-when-sick.html; https://www.cdc.gov/coronavirus/2019-ncov/if-you-are-sick/care-for-someone.html; https://www.cdc.gov/coronavirus/2019-ncov/if-you-are-sick/parents-caregivers.html

¶ https://news.vumc.org/2020/06/30/new-study-examines-coronavirus-transmission-within-households/; https://www.marshfieldresearch.org/News/research-institute-to-study-transmission-of-covid-19-in-households.

** https://www.project-redcap.org/.

†† 95% CIs around the estimated secondary infection rates were calculated using the Wilson method.

§§ 45 C.F.R. part 46.102(l)(2), 21 C.F.R. part 56; 42 U.S.C. Sect. 241(d); 5 U.S.C. Sect. 552a; 44 U.S.C. Sect. 3501 et seq.

¶¶ https://www.cdc.gov/coronavirus/2019-ncov/if-you-are-sick/quarantine.html; https://www.cdc.gov/coronavirus/2019-ncov/hcp/guidance-risk-assesment-hcp.html; https://www.cdc.gov/coronavirus/2019-ncov/community/critical-workers/implementing-safety-practices.html; https://www.cdc.gov/coronavirus/2019-ncov/php/public-health-recommendations.html; https://www.cdc.gov/coronavirus/2019-ncov/hcp/testing-overview.html.
